# [Corrigendum] Changes in expression of p53 and inflammatory factors in patients with ulcerative colitis

**DOI:** 10.3892/etm.2024.12384

**Published:** 2024-01-10

**Authors:** Hui Su, Qian Kang, Haihong Wang, Hui Yin, Linghui Duan, Yuli Liu, Ruying Fan

Exp Ther Med 17:2451–2456, 2019; DOI: 10.3892/etm.2019.7253

Following the publication of the above article, and also an erratum in 2019 (doi: 10.3892/etm.2019.7994) in which an error in the published acceptance date of the article was corrected, the authors have contacted the Editorial Office to explain that they have discovered a couple of further errors in this paper that are in need of correction.

First, in the published version of Fig. 5C on p. 2454, the authors have realized that the β-actin western blots selected for the NCM460 and HepG2 cells were inadvertently the same.The authors have re-examined their original data, and wish to present alternative data for Fig. 5C, as shown in the revised version of Fig. 5 on the next page. Secondly, in the Results section on p. 2455, the “*Detection of p53 expression levels in UC patients and healthy adults*” subsection at the top of the page, the second sentence [“The results revealed that the expression level of p53 in peripheral blood of UC patients (312.16±36.34 ng/l) was obviously decreased compared with that in healthy adults (152.66±44.42 ng/l) (P<0.01) (Fig. 2)”] was written in error. The absolute levels of p53 were incorrectly reported in this sentence based on the detection methods of p53 here, and so the absolute values should not have been stated as such. This sentence should be amended to the following: “The results revealed that the expression level of p53 in peripheral blood of UC patients was obviously decreased compared with that in healthy adults (P<0.01) (Fig. 2)”.

Note that the errors made in the assembly of Fig. 5 and the indicated changes to this sentence in the Results section do not affect the overall conclusions reported in the paper, and all the authors agree with the publication of this corrigendum. The authors are grateful to the Editor of *Experimental and Therapeutic Medicine* for allowing them the opportunity to publish this, and they also apologize to the readership for any inconvenience caused.

## Figures and Tables

**Figure 5:**
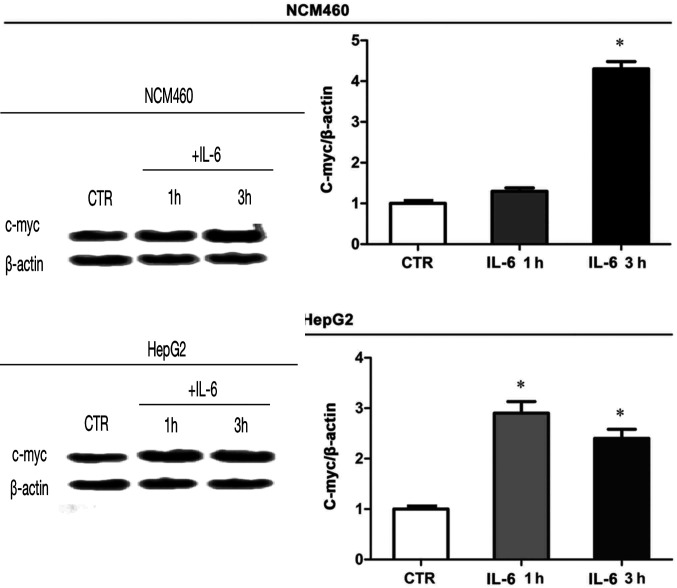
C-myc protein expression in NCM460 and HepG2 cell lines. Analyses of C-myc protein expression in NCM460 and HepG2 cells treated with IL-6 for 1 and 3 h. Results reveal that the C-myc protein expression in NCM460 cells treated with IL-6 for 3 h is higher than that in NCM460 cells treated with IL-6 for 1 h, and higher than that in blank control group (^*^P<0.01). The C-myc protein expression in HepG2 cells treated with IL-6 for 1 and 3 h are significantly increased compared with that in the blank control group (^*^P<0.01), n=3. IL-6, interleukin-6.

